# Toll-Like Receptors and Prostate Cancer

**DOI:** 10.3389/fimmu.2014.00352

**Published:** 2014-07-23

**Authors:** Shu Zhao, Yifan Zhang, Qingyuan Zhang, Fen Wang, Dekai Zhang

**Affiliations:** ^1^Institute of Biosciences and Technology, Texas A&M University Health Science Center, Houston, TX, USA; ^2^Department of Medical Oncology, Affiliated Tumor Hospital of Harbin Medical University, Harbin, China

**Keywords:** toll-like receptor, TLR signaling, prostate cancer, innate immunity, immunotherapy

## Abstract

Prostate cancer is the second leading cause of cancer-related death in men after lung cancer. Immune responses clearly play a critical role in the tumorigenesis and in the efficacy of radiation therapy and chemotherapy in prostate cancer; however, the underlying molecular mechanisms are still poorly understood. Toll-like receptors (TLRs) are a well-known family of pattern recognition receptors that play a key role in host immune system. Recent studies demonstrate that there are links between TLRs and cancer; however, the function and biological importance of TLRs in prostate cancer seems complex. To elucidate the role of TLRs and innate immunity in prostate cancer might provide us with a better understanding of the molecular mechanisms of this disease. Moreover, utilizing the agonists or antagonists of TLRs might represent a promising new strategy against prostate cancer. In this review, we summarize recent advances on the studies of association between TLR signaling and prostate cancer, TLR polymorphisms and prostate cancer risk, and provide some insights about TLRs as potential targets for prostate cancer immunotherapy.

## Introduction

Based on the latest cancer statistics, prostate cancer predictably ranks first among all the cancers in men and second in cancer-related deaths in the United States in 2014 (1). Treatments against prostate cancer, including chemotherapy and radiotherapy, could improve survival; however, many patients will endure relapse and metastasis, which eventually leads to death. These treatments also destroy cancer cells and normal cells alike. Therefore, a more effective and less toxic therapy needs discovery. A promising strategy for dramatically preventing cancer development and improving cancer treatment might rely on immunotherapy. Immune evasion is a hallmark of cancer pathogenesis. Cancer cells escape from immune attack through a variety of mechanisms. A compromised immune system and chronic inflammation increase the incidence of cancer development. Inflammation has been proposed as the seventh hallmark of cancer (2) and an excellent review has elegantly summarized the role of inflammation in prostate cancer development and potential underlying mechanisms (3). Immunotherapy, which utilizes host immune system to fight cancer, has been recently highlighted with several advantages including specificity, less side effects, and less likely to develop resistance. It could be achieved in two ways: stimulating immune system to attack cancer cells or taking away the inhibitory machinery to the immune system in cancer. One potential approach to modulate immune system is targeting pattern recognition receptors (PRRs) in innate immune system, among which toll-like receptors are most well studied.

## Toll-Like Receptor: A Well-Known Family of Pattern Recognition Receptors in Innate Immunity

**Figure 1:**
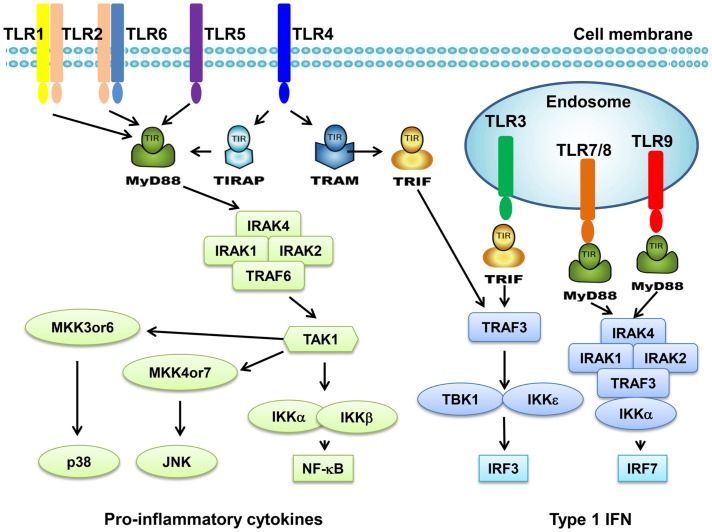
**Toll-like receptors and TLR-mediated signaling pathway**. TLR1 and TLR6 recognize their ligands as heterodimers with TLR2. For TLR4, MD2, and CD14 are required for LPS recognition and signaling. TLR3, TLR4, TLR5, TLR7, and TLR9 are currently thought to deliver their signal by forming homodimers after interacting with their ligands. TLR3, TLR7/8, and TLR9 are intracellular TLRs and are involved in the recognition of nucleic acids. Most TLRs, except for TLR3, signal through MyD88 pathway to activate NF-κB and AP1. TLR3 and TLR4 can signal through MyD88-independent pathway (TRIF pathway) to activate INF-β.

Toll-like receptors are a family of transmembrane receptors that play a key role in the innate immunity. TLRs prevent invading pathogens by recognizing pathogen-associated molecular patterns (PAMPs), which are highly conserved components derived from bacteria, viruses, fungi, and parasites (4, 5). It can also recognize endogenous damage-associated molecular patterns (DAMPs) in different disorders and diseases such as cancer (4, 5). At present, 10 TLRs have been identified in human. TLR1s, TLR2, TLR4, TLR5, and TLR6 are expressed on cell surface; however, TLR3, TLR7, TLR8, and TLR9 are found exclusively within endosomes (Figure 1). Different TLRs exhibit specificity for ligand recognition. TLR2 recognizes bacterial lipoproteins, TLR3 recognizes double-stranded RNA/polyinosinic–polycytidylic acid [poly (I:C)], TLR4 recognizes lipopolysaccharides (LPS), TLR5 recognizes flagellin, TLR7 recognizes single-stranded RNA, and TLR9 recognizes CpG-containing DNA (CpG-ODN) (6–11). TLR10 is so far an orphan receptor and highly expressed in the human spleen (12) and B cells (13). Upon activation, TLRs transmit signals through one or more of four adaptor proteins: myeloid differentiation factor 88 (MyD88), TICAM1 (also known as TRIF), TIRAP (also known as MAL), and TICAM2 (also known as TRAM and TIRP). All TLRs (except for TLR3) and IL-1 receptor family members signal through MyD88. TLR3 signals through TRIF pathway; TLR4 signals through both the MyD88 and the TRIF pathways (4). Stimulation of TLRs leads to activation of NF-κB, MAPKs, Jun N-terminal kinases (JNKs), p38, and ERKs, as well as interferon regulatory factor (IRF3, IRF5, and IRF7) signaling pathways, which results in the production of inflammatory cytokines (14). Activation of TLRs in antigen-presenting cells (APC) also triggers adaptive immunity. TLRs have also been shown to regulate cell death and increase expression of the anti-apoptotic proteins Bcl-2-related protein A1 (BCL2A1), inhibitor of apoptosis 1 (cIAP1), cIAP2, XIAP, and Bcl-2 family members (15).

## TLR Expression and Function in Prostate Cancer

**Figure 2:**
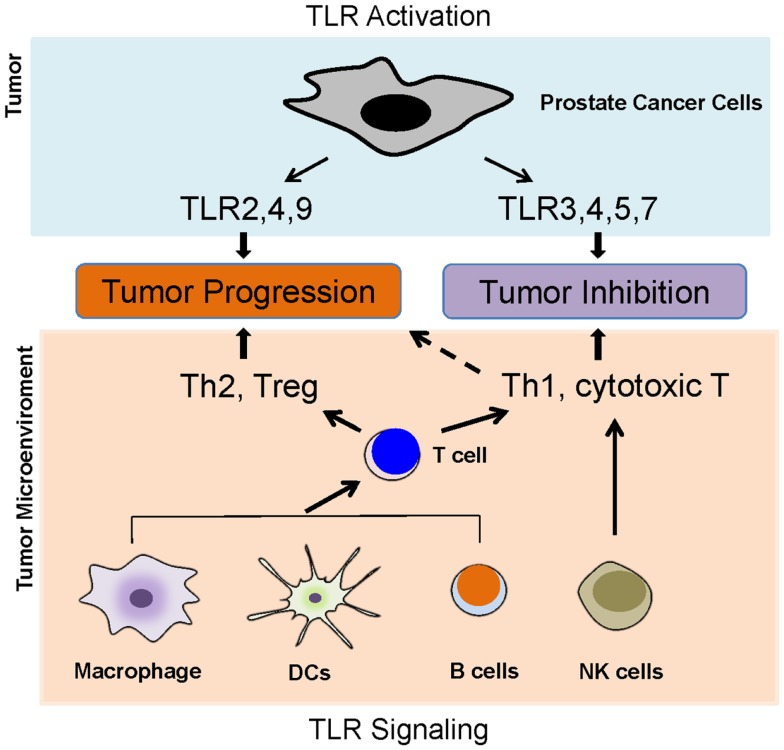
**Toll-like receptors and prostate cancer**. TLR activation in tumor cells and its activation in tumor microenvironment such as in typical innate immune cells lead to a complex scenario, which determines the role of TLRs in prostate cancer development. The activation of TLRs in antigen-presenting cells, such as DCs, macrophages, and B cells, can lead to either Th1 and T cytotoxic responses or Th2 and Treg responses. The activations of TLR2, 4, and 9 in prostate cancer cells appear to promoter tumor growth, but the activation of TLR3, 4, 5, and 7 might inhibit prostate cancer.

Toll-like receptors are predominantly expressed in innate immune cells such as dendritic cells, macrophages, and natural killing (NK) cells. Activation of TLRs in these cells leads to the activation of innate immunity and results in the production of pro-inflammatory cytokines, chemokines, as well as adhesion molecules, and then facilitates the activation of adaptive immunity (16). Intriguingly, growing evidence has demonstrated that TLRs are also expressed in tumor cells. TLR activation in tumor cells and its activation in tumor microenvironment such as in typical innate immune cells lead to a complex scenario (Figure 2); therefore, the activation of TLRs might play a “double-edged sword” role in the influence of tumor progression.

In most cases, it is difficult to figure out a specific pathogen to activate TLR signaling in prostate cancer. An endogenous TLR ligand, DAMPs released from damaged and/or necrotic tissues, might play a pivotal role. In term of endogenous TLR ligands in cancer, HMGB1 can activate TLR2 and TLR4 (17), and versican acts as a TLR2 agonist (18). Peroxiredoxin 1 (Prx1) appears to be an agonist of TLR4 in prostate cancer development (19). Perhaps, there are more endogenous TLR ligands that need to be further identified and verified.

The activation of some TLRs might prevent the tumor growth of prostate cancer (Figure 2). It has been shown that TLR3 is expressed in prostate cancer cells (20–25). TLR3 mRNA is detected in three prostate cancer cells lines including LNCaP, PC3, and DU-145. TLR3 mRNA level was clearly enhanced in prostate cancer cells by stimulating with poly (I:C), which suggests a functional role of TLR3 in prostate cancer (20). TLR3 protein was also expressed at similar levels in LNCaP and DU-145 cells, with a slightly lower expression in PC3 cells. Treatment with poly (I:C) rapidly triggered NF-κB-dependent expression of inflammatory molecules. Condition medium from poly (I:C)-treated LNCap and DU145 cells recruited leukocyte subpopulation, indicating that TLR3 activation might influence early immune responses in tumor microenvironment (25). Stimulation with poly (I:C) strongly suppressed prostate tumor growth *in vivo*, perhaps due to increased infiltration of T lymphocytes and NK cells in a type I IFN-dependent manner (24). In human prostate cancer patients, 85 in 112 prostate carcinomas samples showed positive expression of TLR3. High TLR3 expression level was significantly associated with high probability of the recurrence of prostate cancer (23). Paone and colleagues found that TLR3 could regulate the process of angiogenesis and apoptosis in prostate cancer cells through hypoxia-inducible factor 1α (HIF-1α) and PKC-dependent mechanism (21, 22). TLR5 is expressed in LNCap and DU-145 by which stimulation triggers the production of chemokines that recruit immune cells, including NK cells and cytotoxic CD8 cells, which most likely contribute to tumor inhibition (25).

The activation of other TLRs might play a different role in the tumor growth of prostate cancer (Figure 2). The expression of TLR4 in prostate cancer has been demonstrated in several animal models. Studies revealed a constitutive expression of TLR4 in the epithelial cells of rat ventral prostate as well as in a rat adenocarcinoma cell line and in prostate primary culture cells (26, 27). TLR4 is also expressed in DU-145, PC3, and normal prostate gland in both stroma and epithelium (28, 29). In addition, TLR4 has also been shown to be expressed in clinical samples of prostate cancer. Initially, TLR9 expression was thought to be restricted to immune cells, but recent studies have showed that a variety of tumor cell types including prostate cancer also express functional TLR9 (23, 30, 31). A clinical study demonstrated that TLR9 is expressed in prostate cancer specimens (23). Joanna et al. found that TLR9 is expressed in human prostate cancer cell lines LnCaP, C4-2B, Du-145, PC3, and in clinical samples of prostate cancer through immunohistochemistry and western blotting, but not in MDA Pca2b and stromal cells of the clinical adenocarcinoma samples (32). TLR9 expression was also statistically significantly increased in prostate cancer epithelium and stroma, compared with the same cellular compartments in benign hyperplasia, especially in the most poorly differentiated forms (30).

The function and biological importance of TLRs in prostate cancer seems complex (Figure 2). Perhaps the distinct and unidentified TLR signaling pathways are activated in cancer cells or innate immune cells during tumor progression; or, the first activation of TLR in cancer cells or innate immune cells markedly affect the subsequently activation and induced effectors. The mystery will be further investigated and will affect the potential of TLR agonists or antagonists as anti-tumor therapeutic agents.

## MicroRNA Regulate TLRs in Prostate Cancer

MicroRNAs (miRNAs) are a class of small non-coding RNAs (~22 nt in length), which negatively regulate gene expression at the post-transcriptional level (33). By binding to target sequences within the 3′ UTR of mRNA, miRNAs induce gene silencing by either inhibiting translation or leading to degradation of mRNA. MiRNA alterations are shown to be involved in both initiation and progression of human cancer (34–39). Deregulation of miRNAs is implicated as an important mechanism in tumorigenesis and several miRNAs have been proposed as oncogenes or tumor suppressors (40–42).

MicroRNAs are emerging as a fundamental mechanism in the regulation of TLR signaling (43–47). Recent works have linked miRNAs and TLRs in prostate cancer. MiR-29a has been shown as a potential tumor suppressor miRNA to regulate TRAF-4 expression in metastatic prostate cancer (48). TLR3 activation by poly (I:C) induces upregulation of miRNAs including miR-29b, -29c, -148b, and -152, which target DNA methyltransferases and leads to reexpression of oncosuppressor RARβ in prostate cancer cells (49). TLRs activation facilitates either prostate cancer inhibition or progression. MiRNAs are likely to act as important regulators to control TLRs expression and signaling, thus contribute to prostate cancer development.

## TLR Signaling in Prostate Cancer

Toll-like receptor signaling pathway has been well defined in innate immune cells. TLR ligation recruits one or more adaptor proteins such as MyD88, TRIF, Mal, and TRAM though TIR domain interactions. Most TLRs except TLR3 go through a MyD88-dependent signaling pathway. MyD88 engagement activates IL-1 receptor associated kinase (IRAK), which interacts with tumor necrosis factor receptor associated factor 6 (TRAF6), resulting in the activation of MAPK and NF-κB signaling. TLR3 and TLR4 activate a MyD88-independent signaling pathway. TRIF is recruited upon stimulation and leads to the activation of NF-κB and type I IFN signaling.

Although TLR3 can be activated in prostate cancer cells, the molecular signaling pathway has not been fully elucidated. A recent study in human prostate cancer cells suggests that TLR3 signaling triggers apoptosis and growth arrest of LNCaP cells partially through inactivation of the PI3K/Akt pathway. CyclinD1, c-Myc, p53, and NOXA are indicated to play a role in poly (I:C)-treated LNCaP cells (20). In other studies, HIF-1α facilitates apoptosis through a PKC-dependent mechanism in poly (I:C)-treated prostate cancer cells. TLR3 activation by poly (I:C) activates JNK and p38 through PKC-α and triggers apoptosis in a caspase-8 dependent manner (21, 22). In LNCap cells, poly (I:C) treatment upregulates a pattern of chemokines, including CCL3, CCL4, CCL5, CCL8, CXCL9, and CXCL10, which could induce massive NK cell and CD8 T cell chemotaxis. Moreover, poly (I:C) induced the expression of inflammatory molecules such as IL-6 and IL-12, which are NF-κB signaling dependent (25). In TRAMP tumor model, poly (I:C) treatment recruits NK cells and T lymphocytes through a type I IFN dependent mechanism, resulting in suppression of tumor growth (24). TLR5 agonist flagellin can activate NF-κB signaling in LNCaP and DU145 cells, and lead to the production of pro-inflammatory molecules (25).

Stimulation of TLR4 in DU145 by LPS activates NF-κB signaling pathway, which leads to production of pro-inflammatory cytokines such as IL-6 and IL-1β through MyD88-dependent pathway (29). In addition, TLR4 activation increases expression of VEGF and TGF-β1 in PC3 cells, which promote tumor development (28). Also, knockdown of TLR4 using siRNA in PC3 cells reduces tumor cell migration and invasion (50). TLR9 stimulation by CpG-ODN plays an important role in prostate cancer invasion. This effect is mediated by activating NF-κB and upregulation of COX-2 (31). TLR9 expression in prostate cancer cells has similarly been found to enhance invasiveness via induction of MMP-13 *in vitro* (32). In both studies, CpG-ODN stimulation did not affect cellular proliferation, which suggests TLR9 signaling plays a role in cancer progression and metastasis.

These defined TLR signaling pathways seem difficult to help understand why the activation of some TLRs such as TLR3 inhibits tumor growth but the activation of other TLRs such as TLR2 promotes tumor growth (Figure 2). Some distinct TLR signal pathways must exist to determine the specific effectors in the different TLR activations leading opposite consequences.

## TLR Gene Polymorphisms and Prostate Cancer Risk

Polymorphisms in TLR genes are reportedly related to susceptibility of a large spectrum of infectious and inflammatory diseases. Growing evidence suggest that chronic intra-prostatic inflammation contribute to prostate cancer progression. It was suggested that TLR gene polymorphisms might alter TLR signaling, thus affecting inflammation and prostate cancer risk. A number of studies have been done to investigate whether there is a connection between TLR gene polymorphisms and prostate cancer risk, and the results are controversial (51, 52).

Single nucleotide polymorphisms (SNPs) in TLR4 were reported to be associated with prostate cancer risk in several studies (53–58). Sequence variants in TLR gene cluster (TLR6-TLR1-TLR10) were also reported to be associated with prostate cancer risk (51, 52). However, controversial results were also obtained. Shui and colleagues investigated 10 SNPs in TLR4 and found no significant correlation between TLR4 genetic variation and prostate cancer risks (59). Chen et al. reported that sequence variants of gene cluster TLR6-TLR1-TLR10 were not associated with the risk of prostate cancer (60). A meta-analysis by Lindström et al. did not show clear correlation between TLR gene polymorphisms and prostate cancer risks.

The discrepancies among these results might be due to multiple factors including detection method, the race of population, and sample size. It is important to clarify this issue because it will determine not only whether the TLR polymorphisms can be used as a diagnosis/prognosis marker but also whether we can develop a novel strategy to treat prostate cancer by targeting TLRs and their signaling pathway. A more comprehensive study including a sufficient sample size should be performed to investigate the association between TLR gene polymorphisms and prostate cancer risk.

## Targeting TLRs for Prostate Cancer Immunotherapy

The ability of TLRs to manipulate prostate cancer development has raised the interests in developing immunotherapy against prostate cancer with the TLR agonists or antagonists. Actually, three drugs targeting TLRs have been approved by FDA for use in cancer patients: the bacillus Calmette–Guérin (BCG), monophosphoryl lipid A (MPL), and imiquimod (61). BCG is prepared from an attenuated strain of *Mycobacterium bovis* and activates TLR2/4. BCG is used as a vaccine in prevention of tuberculosis, but also for treatment of *in situ* bladder carcinoma. Derived from LPS as a potent TLR4 agonist, MPL is an active component of Cervarix, which is used against cancer-causing human papillomavirus (HPV) (62, 63). Imiquimod, one of the most successful drugs targeting TLRs, is a synthetic imidazoquinoline that signals though TLR7 and is commonly used in the treatment of skin cancer such as basal cell carcinoma and Bowen’s disease (64–66). Imiquimod induces the proinflammatory cytokines including IFNα, IL-6, and TNF-α (67). The activation of TLR7/8 leads to a Th1 response and an anti-tumor activity, which depends on IFNγ (68). In prostate cancer, to support this concept, Han et al., reported that Imiquimod can inhibit both human and mouse prostate cancer growth by inducing apoptosis (69, 70).

A number of preclinical and clinical studies are ongoing to investigate the immunotherapeutic potency utilizing TLRs against prostate cancer. TLR3 activation directly triggers apoptosis of human prostate cancer cells (21); therefore, TLR3 agonists have potential to be developed as anti-tumor therapeutic agents. Indeed, Ampligen, composed of poly (I:C) (a TLR3 agonist), has been shown to inhibit a variety of tumor growth in early clinical trials (71, 72). Hiltonol, a particular formulation of poly (I:C), is currently in Phase I/II clinical trial to evaluate its safety and efficacy (71). Meanwhile, a phase 2 clinical study (NCT00514072) utilizing a BCG vaccine to treat prostate cancer is ongoing. A multi-peptide, dual-adjuvant telomerase vaccine (GX301) in which Imiquimod is an active component showed less toxic and highly immunogenic in prostate cancer patients, but requires future studies to determine its clinical efficacy (73). Furthermore, TLR4 stimulation by LPS is shown to contribute to chemoresistance to docetaxel in prostate cancer cells (74).

## Concluding Remarks

Toll-like receptors play a critical role in innate immunity. TLRs are expressed not only in innate immune cells, but also in non-immune cells including cancer cells. Functional expression of TLRs has been linked to prostate cancer development. TLRs may serve as a double-edged sword in prostate cancer tumorigenesis by promoting malignant transformation of epithelial cells and tumor growth, or on the contrary, inducing apoptosis, and inhibiting tumor progression. The consequences might be dependent on complex signaling networks triggered by TLRs activation and tumor microenvironment. Genetic variations and polymorphisms of TLRs have been associated with prostate cancer; however, the results are inconclusive and need further validation (75, 76). The ability of boosting immune responses but with less serious side effect makes TLRs a good target to treat cancers. A wave of preclinical and clinical studies showed the potential of developing treatment targeting TLRs against prostate cancer. Based on these researches, one of the most probable approaches is to use agents targeting TLRs as adjuvants along with other treatments (67, 68, 71, 77, 78). Above all, elucidation of the mechanisms of cancer cell TLR signaling and crosstalk with other signaling pathways as well as the mechanisms of cancer progression will definitely provide a promising novel strategy for cancer treatment.

## Conflict of Interest Statement

The authors declare that the research was conducted in the absence of any commercial or financial relationships that could be construed as a potential conflict of interest.
